# Coexistence of perfect spin filtering for entangled electron pairs and high magnetic storage efficiency in one setup

**DOI:** 10.1038/srep24417

**Published:** 2016-04-14

**Authors:** T. T. Ji, N. Bu, F. J. Chen, Y. C. Tao, J. Wang

**Affiliations:** 1 Department of Physics and Institute of Theoretical Physics, Nanjing Normal University, Nanjing 210023, China; 2 Department of Physics, Southeast University, Nanjing 210096, China

## Abstract

For Entangled electron pairs superconducting spintronics, there exist two drawbacks in existing proposals of generating entangled electron pairs. One is that the two kinds of different spin entangled electron pairs mix with each other. And the other is a low efficiency of entanglement production. Herein, we report the spin entanglement state of the ferromagnetic insulator (FI)/*s*-wave superconductor/FI structure on a narrow quantum spin Hall insulator strip. It is shown that not only the high production of entangled electron pairs in wider energy range, but also the perfect spin filtering of entangled electron pairs in the context of no highly spin-polarized electrons, can be obtained. Moreover, the currents for the left and right leads in the antiferromagnetic alignment both can be zero, indicating 100% tunnelling magnetoresistance with highly magnetic storage efficiency. Therefore, the spin filtering for entangled electron pairs and magnetic storage with high efficiencies coexist in one setup. The results may be experimentally demonstrated by measuring the tunnelling conductance and the noise power.

Entanglement of quantum states, a kind of nonlocal quantum correlation, is required in many applications of quantum information^1^,^2^,^3^. However, in so-called Entangled electron pairs superconducting spintronics, there still exists huge challenges. For existing proposals of generating entangled electron pairs, the two kinds of different spin entangled electron pairs mix with each other, and the efficiency of entanglement production is low. A spin entangled electron pair is usually much less sensitive to decoherence than an orbitally entangled one^4^, and thus the former is a better candidate in experiments^5^. The experimental manipulation, i.e., the controlled production and detection of entangled electron pairs, still remains a challenge due to the dirty environment in solids^5^,^6^, which can easily ruin the entanglement signals. Since Cooper pairs consist of two electrons that are both spin and momentum entangled, superconductors (SCs) are deemed as natural sources for entangled electrons^7^,^8^,^9^,^10^. A Cooper pair can be spatially deformed by the inverse nonlocal Andreev reflection (AR)^8^,^11^, a process which emits two spin entangled electrons in two conventional metallic leads where they can be probed separately. A straightforward way to observe the nonlocal AR is by nonlocal conductance measurements^12^,^13^. However, this method has the drawback that the signatures of nonlocal AR are often completely masked by another nonlocal process known as electron elastic cotunneling^6^,^14^, which does not involve Cooper pairs and is therefore a parasitic process. Two requirements for experimental creating of spin entangled electron pairs on solid state spins are necessary^5^. One is highly spin-polarized electrons, and the other is a perfect correlation measurement, i.e., two spin entangled electrons traveling through two different paths can reach their respective receivers with a high detection rate. Nevertheless, the usual spin filter can destroy the spin entanglement of the underlying Cooper pair and half-ferromagnetic metals cannot give rise to nonlocal AR.

Recently, the quantum spin Hall (QSH) system has been proposed as the promising creator and detector for entangled electron pairs^15^. The QSH state, a kind of two-dimensional (2D) topological insulator, has recently been predicted and observed in mercury telluride quantum wells^16^,^17^,^18^,^19^. The transport of the QSH insulator is characterized by gapless helical edge states, which are topologically protected by the time reversal symmetry. The spin-up and spin-down carriers of the same edge in the helical edge states move strictly toward opposite directions^16^. The two edges in a narrow QSH strip can couple together to produce a gap in the energy spectrum^20^. A normal metal (NM)/SC/NM junction on a QSH strip was investigated^15^. The nonlocal AR is totally suppressed in absence of interedge coupling due to the helicity conservation of the carriers^21^, while occurs in the presence of interedge coupling^22^,^23^. Under the resonant bias voltage, the local AR, the elastic cotunneling, and the normal reflection can be all suppressed by proper tuning of the band structures in the normal leads^15^. All incident electrons can be converted into holes in the other terminal, indicating the nonlocal AR of 100% fraction. However, there exist few energy values corresponding to the 100% fraction, being unfavorable for application. More importantly, the entangled electron pairs with two kinds of spins are mixed together, which is not just expected.

In this paper, we propose a topological ferromagnet insulator (FI)/*s*-wave SC/FI hybrid structure based on a narrow QSH strip, in which the interplay of the ferromagnetism and interedge coupling is exhibited. In the proposed setup, the detection of spin entangled electrons can be achieved efficiently by proper tuning the bias voltage and exchange field in the two FI regions. It is found that, for nearly all energies in the energy window, the nonlocal AR for one spin in the ferromagnetic (F) alignment occurs at a much great fraction, while is thoroughly suppressed for the other opposite spin. This means that the entangled electron pairs with only one kind of spin can be obtained, and thus this setup can be used as the creator and spin filter of entangled electron pairs. Furthermore, in the antiferromagnetic (AF) alignments, nonlocal AR probability in the energy window is zero and the currents for the left and right leads are both zero, which indicates tunnelling magnetoresistance of 100% with highly magnetic storage efficiency. Therefore, the setup can unite the two functions in one device, and is favorable to handling the quantum information and magnetic storage at the same time. The properties of not only the conductance but also the noise to probe the nonlocal AR process are also investigated.

## Concurrence of Ferromagnetism and interedge coupling

**Figure 1:**
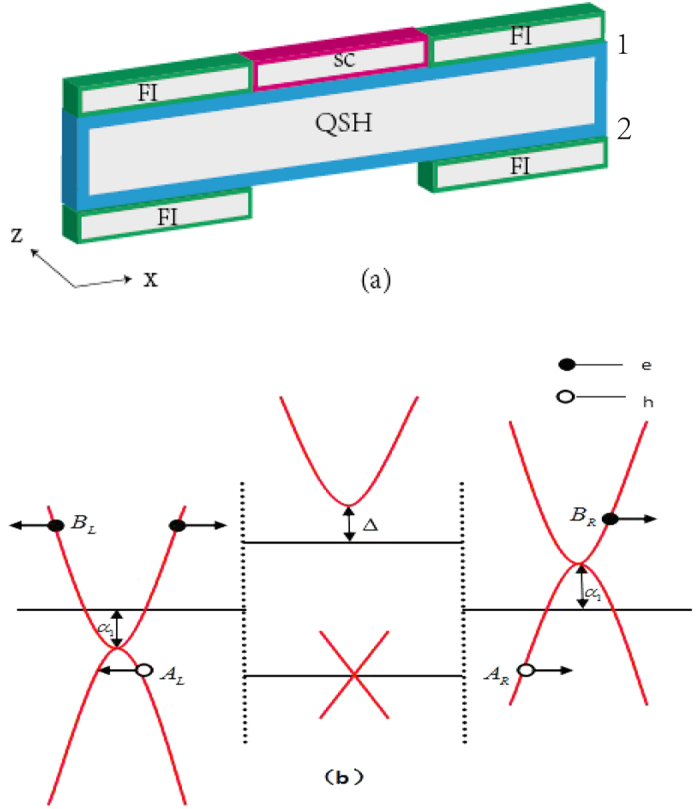
Schematics of the proposed structure and the corresponding energy band. (**a**) The QSH strip with two edges (edges 1 and 2) is sketched as the blue bar.The direction of the growth direction is along *z*-axis and the carriers in the two edges move along *x*-axis. An *s*-wave SC is deposited on one edge of the QSH strip (edge 1) in the middle region (0 < *x* < *d*), and four identical FIs on the two edges in the left and right regions. (**b**) The energy band of the structure is depicted as the red lines. The filled circles stand for the incident electron with probability 1, the reflected electron with probability *B*_*L*_, and the transmitted electron with probability *B*_*R*_, respectively. And the open circles are respectively the local and the nonlocal Andreev reflected holes with probabilities *A*_*L*_ and *A*_*R*_.

We consider a QSH strip shown in Fig. 1(a) with the longitudinal direction along the *x* axis. An *s*-wave SC is deposited on one edge of the QSH strip (edge 1) in the middle region (0 < *x* < *d*)^21^, and four identical FIs on the two edges in the left and right regions. Due to the proximity effect, superconductivity and ferromagnetism are exhibited in the contacting areas of two edges^21^,^24^. The superconducting gap can penetrate into a QSH system with only a few atomic layers^25^, and thus only the edge of the QSH strip in touch with the SC (edge 1) can be assumed superconducting and the other edge (edge 2) remains normal. Electron wave functions have overlaps within the narrow strip width, leading to the two edges of the QSH strip coupled together. For the left and right regions, the coupling strength *α*(*x*) of the two complex ferromagnetic (CF) edges can be taken as *α*_1_ without FIs. However, for the middle region, the wave functions of electrons in the QSH strip can penetrate into the bulk SC^26^, inducing the lower interedge coupling strength *α*_2_.

Here, the spin-flip scattering is briefly discussed. Because of the effect of the FIs, the states of the two edges in the corresponding regions are not of time-reversal symmetry, which is different from those protected in NM/SC/NM junction of ref. 15. And thus the states are not protected so that spin-flip may be induced. However, the exchange field in the two edges are caused by the proximity of the FIs, which perhaps gives to the spin-flip scattering but it is very weak and cannot be considered. Furthermore, in the finite size system, since the edge states are not well separated, electron scattering between the edge states at the two edges become possible. For QSH states, the two states at the opposite edges have opposite spins. Therefore, when they couple with each other, the spin-flip scattering also can occur. But as shown in ref. 20, electron wave function overlaps almost in the middle part of narrow stripe far from the two edges, which leads to the two edges of the strip coupled together. As a result, the spin-flip scattering induced by the coupling in the two edges is also very weak and can be similarly ignored as in ref. 15. The effect of the energy gap caused by the coupling on the result is much greater than that of the spin-flip scattering.

The eight-component wave function 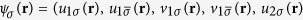, 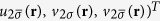 with spin 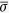 opposite to *σ* can be applied for this system. The Bogoliubov-de Gennes (BdG) equation^27^ is given by 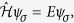 where *E* is the quasiparticle energy relative to the Fermi energy *E*_*F*_ and


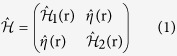


with


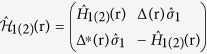


and 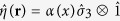. Here, the pair potential 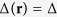 in edge 1 of the SC region and 0 in the two CF regions, 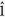 is the 2 × 2 unit matrix, 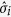 (*i* = 1, 2, 3) denote the Pauli matrices in spin space, and 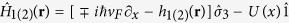 with *v*_*F*_ being the Fermi velocity is the Dirac-like Hamiltonian for helical particles in the two edges. The positive (negative) sign is for spin-up (down) electrons in edge 1(2), the potential *U*(*x*) in the three different areas can be tuned by the gate voltage or doping independently, and *h*_1(2)_(**r**) denotes the exchange field. The four CF edges have the same exchange field, which is described by 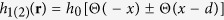, where the plus and minus signs respectively correspond to the F and AF alignments of the left and right CF regions.

The energy spectra in the left and right CF regions are written as 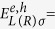 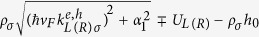, where *ρ*_*σ*_ is 1 (−1) for spin-up (down) channel, *U*_*L*(*R*)_ stands for potentials, 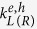 denote wave vectors, and a gap of 2*α*_1_ is produced by the coupling of the two edges^20^. The parabolic-like dispersion relations at the band bottom or top are depicted in Fig. 1(b), where *E*_*F*_ can cross the different positions of the energy band by the tuning the potentials applied to the left and right regions^28^,^29^.

Since spin-flip effect is ignored, the spin-up channel and the spin-down one can be considered independent, and thus the BdG equation is decoupled into two sets of four-component equations with four-component wave function 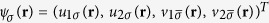 for spin-*σ* channel^27^. The solutions of the BdG equation in the left, the middle, and the right areas are obtained as


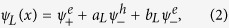



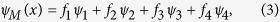



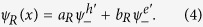


Here, 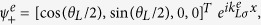 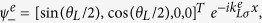

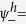
 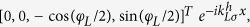 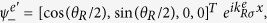 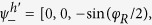

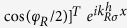
, and 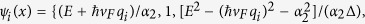

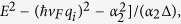
 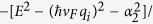 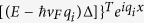 (*i* = 1−4), where 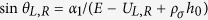, 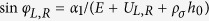, the wave vectors for electron and holes in the CF regions and SC region are respectively given by 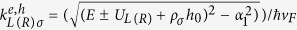, and 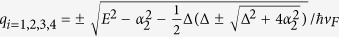. The coefficients *a*_*L*_, *a*_*R*_, *b*_*L*_, and *b*_*R*_ in Eqs (2) and (4) correspond to the local AR, nonlocal AR, normal reflection, and elastic cotunneling, respectively. All the coefficients *a*_*L*_, *a*_*R*_, *b*_*L*_, *b*_*R*_, and *f*_*i*_(*i* = 1−4) will be determined by the following matching boundary conditions


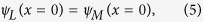



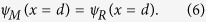


The probabilities of four tunneling processes are respectively given by 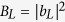, 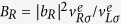, 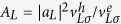, and 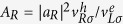 with *A*_*L*_ + *A*_*R*_ + *B*_*L*_ + *B*_*R*_ = 1, where 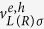 are the velocities of the quasiparticles.

## Results

### High production rate of entangled electron pairs

**Figure 2:**
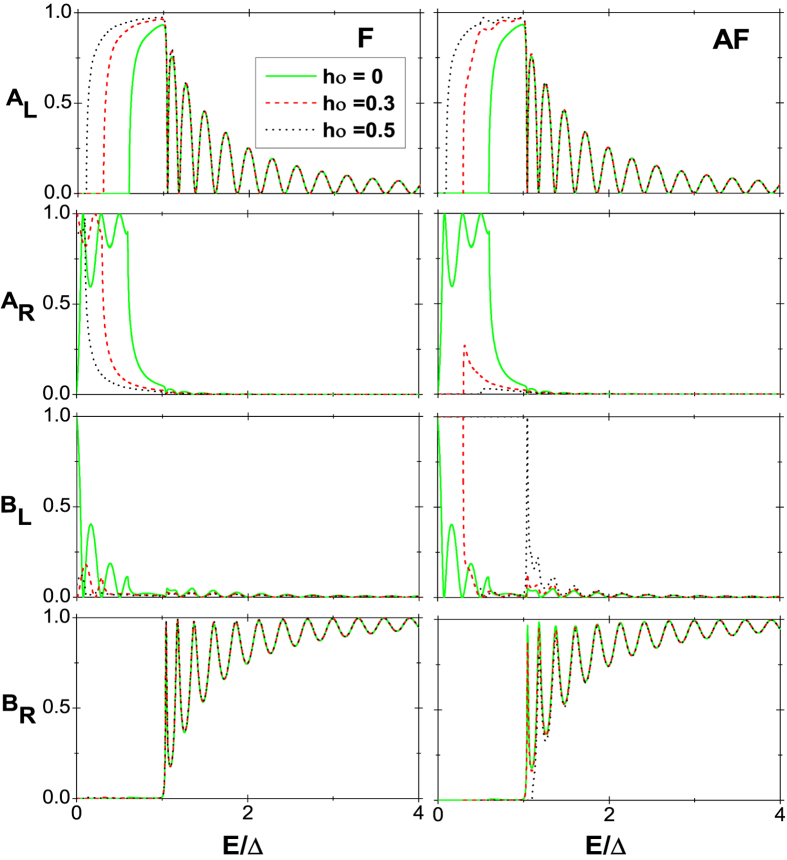
Probabilities of the four tunneling processes as a function of energy for the incident spin-up electrons in the F (the left column) and AF (the right column) alignments. Here, *α*_1_ = 0.3, *α*_2_ = 0, *U*_*L*_ = −*α*_1_, and *U*_*R*_ = *α*_1_.

In order to acquire some transparent physical insights, we first investigate the simple case for *α*_2_ = 0, and then *α*_2_ = *α*_1_ as in ref. 15. Here, we take the units 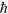
*v*_*F*_ = 1 and Δ = 1, and set *d* = 10 *ξ* with 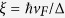 being the superconducting coherent length. In Fig. 2, the probabilities of the various tunneling processes for incident spin-up electrons in the F and AF alignments are respectively shown as a function of *E* at different exchange field *h*_0_, where *α*_1_ = 0.3, *α*_2_ = 0, *U*_*L*_ = −*α*_1_ and *U*_*R*_ = *α*_1_. In the case of *h*_0_ = 0, the CF edges turn to normal ones and the results for the F and AF alignments, as shown by the solid (green) lines in Fig. 2, are thoroughly identical, which is just that in ref. 15. In the energy window 0 < *E* < 2*α*_1_, there are not only no holes propagating in the left region but also no electrons propagating in the right region, indicating that the local AR and elastic cotunneling are forbidden for particles of energy lower than 2*α*_1_. Thus the current is completely contributed from the nonlocal AR process. However, when *h*_0_ ≠ 0 as in refs 30, 31, 32, the situation becomes much different. In the F alignment, for instance, at *h*_0_ = 0.3, the bottom of the spin-up conduction subband touches the top of the spin-up valence subband in both the left and right CF edges. And the Fermi level is *α*_1_ higher than the bottom of the spin-up conduction subband in the left region while the case is just contrary in the right region as shown in Fig. 1(b). Therefore, the energy window turns into 0 < *E* < *α*_1_ and it follows that with the increase of *h*_0_, the energy window turns narrower. More importantly, although one can find from Fig. 2 that the fraction of the nonlocal AR at the peaks in the energy window can be as high as 100% no matter if *h*_0_ is zero, the degree of oscillation turns weaker with increasing *h*_0_, i.e., the difference between the peak and valley becomes small. This stems from that, the mismatches of wave vectors between the left or right and the middle regions are diminished with the enhancement of exchange field *h*_0_ in the CF regions^15^. The large mismatch can be deemed as a strong barrier at the interface, which plays an important role in degree of oscillations. At the peaks of the curves during the energy window, all the incident electrons are converted into holes on the right side through the nonlocal AR, which can be used to generate entangled electron pairs separately with 100% efficiency, however, the peaks are too few. Hence, by increasing *h*_0_, we can create entangled electron pairs separately for all energies in the energy window with very high efficiency due to the weaker oscillation.

### Coexistence of perfect spin-filtering for entangled electron pairs and magnetic storage with high efficiency

**Figure 3:**
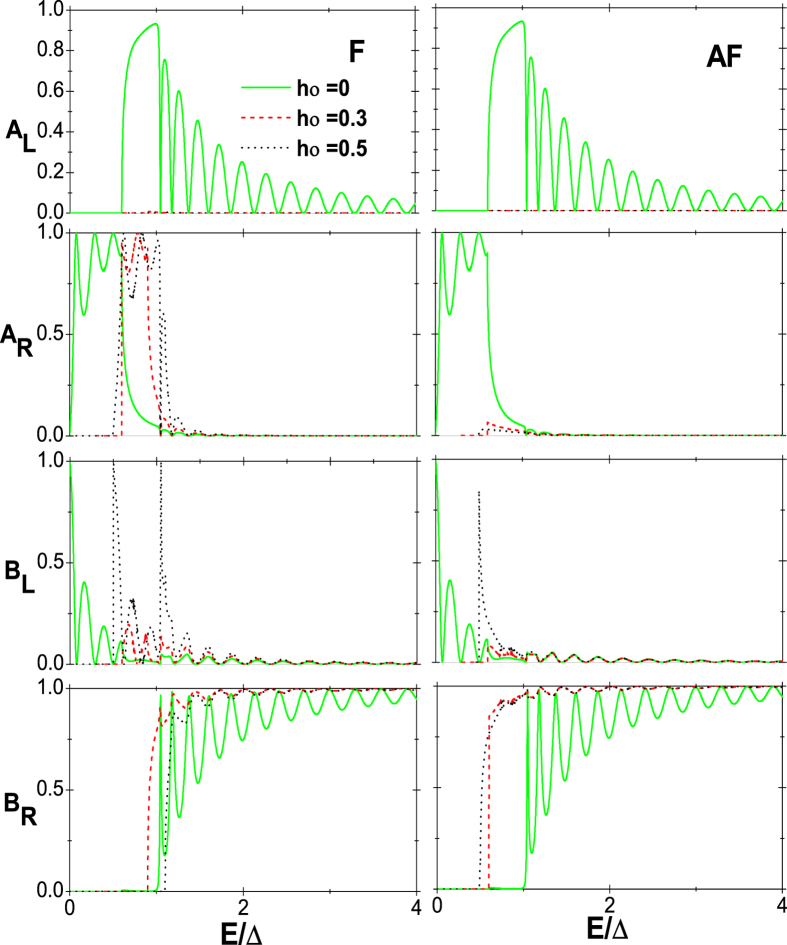
Fig. 2 except for the incident spin-down electrons.

**Figure 4:**
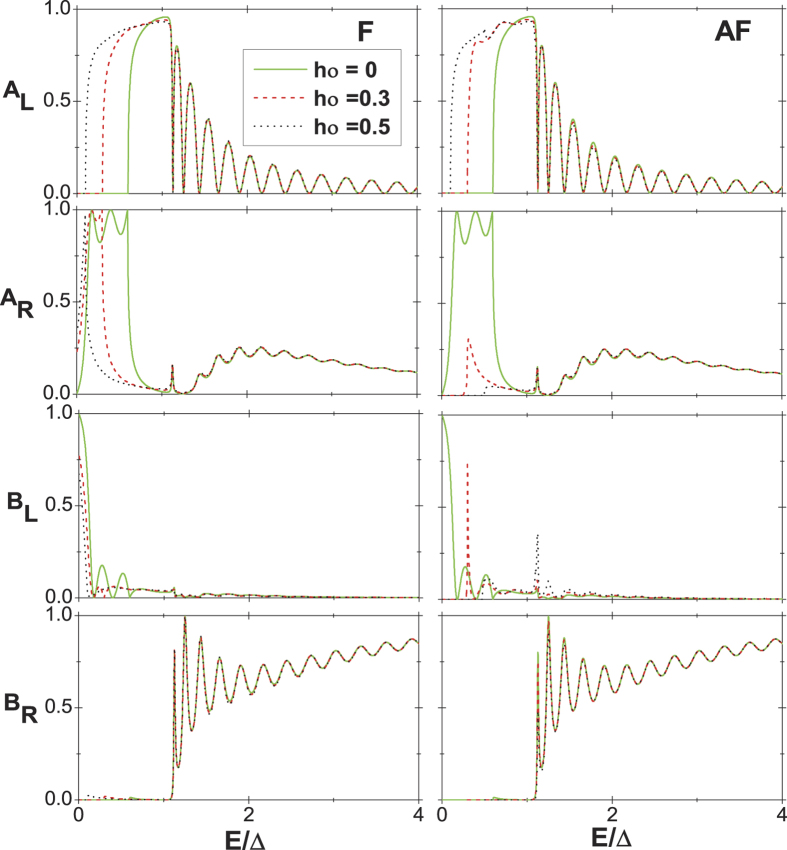
Probabilities of the various tunneling processes as a function of energy for the incident spin-up electrons in the F (the left column) and AF (the right column) alignments. The same parameters as in Fig. 2 except that *α*_2_ = *α*_1_.

As for the AF alignments, the Fermi level at *h*_0_ = *α*_1_ is 3*α*_1_ lower than the bottom of the spin-up conduction subband and *α*_1_ higher than the top of the spin-up valence subband in the right region. This means that in the energy window 0 < *E* < *α*_1_, there are no holes propagating in the left region and no electrons and holes propagating in the right region. As a result, in this energy window, the local AR, nonlocal AR, and elastic cotunneling are forbidden for particles of energy lower than *α*_1_. The current continuity condition becomes *B*_*L*_ = 1, and thus the current is zero according to the following current formalism, which is much different from that in the F alignment. It follows that, at *h*_0_ ≠ 0, neither the entangled electron pairs nor current for spin-up electron can be probed in the left and right regions in the corresponding energy window for the AF alignment. Next, the four probabilities for incident spin-down electrons are also presented, as shown in Fig. 3. At *h*_0_ = 0, the results are the same as those for incident spin-up electrons, which is natural. However, as *h*_0_ = *α*_1_, in the energy window 0 < *E* < 2*α*_1_, *A*_*R*_ = *B*_*L*_ = 0, implying that there are also no electrons propagating in the left region and no holes propagating in the right region. The features are still ascribed to the interplay of ferromagnetism and interedge coupling. More specially, the modification of the gap induced by them for the spin-down subband is much different from that for the spin-up subband. This can be also embodied by the wave functions in Eqs (2) and (4) for the incident spin-down electrons being much different from those for the incident spin-up ones. With the increase of *h*_0_, in the F alignment, the energy window for no holes propagating in left region and no electrons propagating in the right region gets wider, however, the one for no electrons propagating in the left region and no holes propagating in the right region turns slightly wider due to the interedge coupling. In the context of AF alignment, with increasing *h*_0_, the energy windows for the four tunneling processes being forbidden all turn a little narrower. These are attributed to the combination of the spin-down electron incidence with the interedge coupling and AF alignment. Therefore, we can conclude that in a certain energy window, four tunneling processes for the incident spin-down electron are all forbidden in the A alignment, and can obtain 100% spin polarized current in the F alignment and zero current in the AF alignment. We also give the results for the case *α*_2_ = *α*_1_ for the incident spin-up electrons in Fig. 4. It is shown that the interedge coupling in the middle region does not alter the tunneling behaviors qualitatively. For the incident spin-down electron, the behaviors are not also significantly changed, which is not given by figures for simplicity. These indicate that the nonlocal AR is not very sensitive to the variation of *α*_2_.

### Differential Conductances and Zero-frequency Noise Power

In what follows, we investigate the tunneling conductance of the proposed structure, which can reflect the characteristics of the four tunneling processes. A bias voltage *eV* is applied to the left lead. The right lead and the SC are grounded^15^. The positive direction is assumed as the current flowing into the SC, then the currents in the left and right leads at zero temperature are obtained in accordance with the Blonde-Tinkham-Klapwijk (BTK) theory^33^


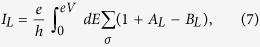



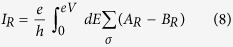


**Figure 5:**
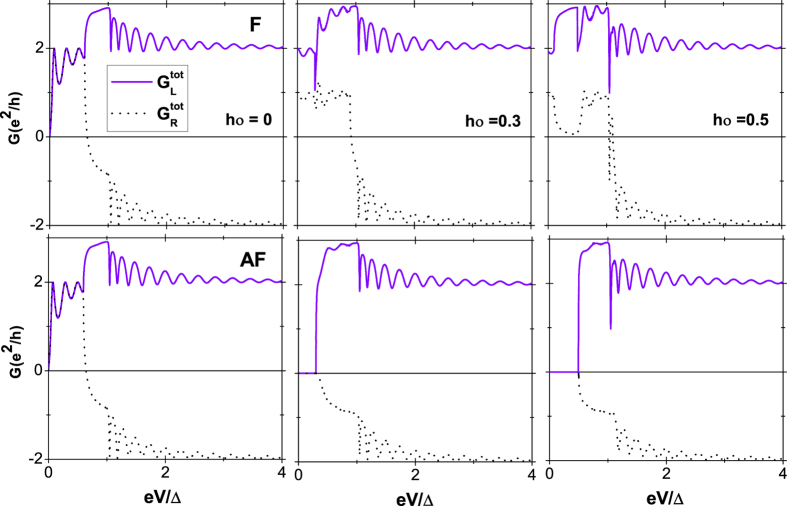
The different conductances 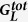 and 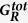 in the F (upper panel) and AF (lower panel) alignments as a function of the bias voltage *eV*. The same parameters as in Fig. 2.

**Figure 6:**
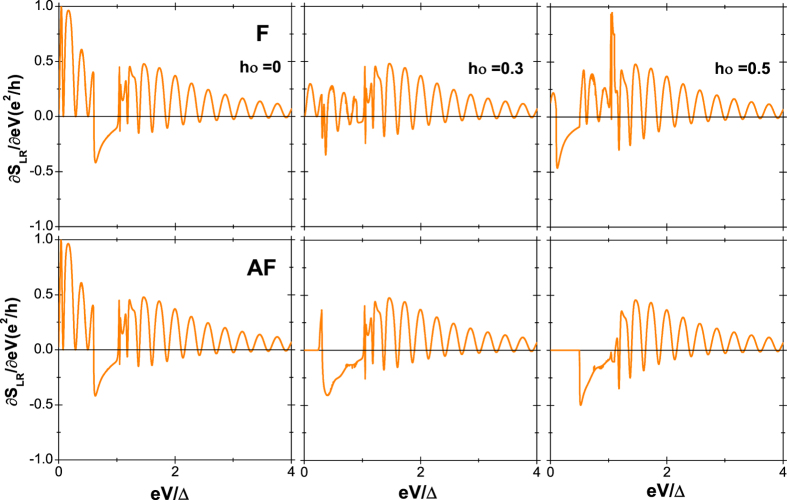
The noise response in the F (upper panel) and AF (lower panel) alignments as a function of the bias voltage *eV*. The same parameters as in Fig. 2.

The total differential conductances from the spin-up and -down channels in the left and right leads are written as


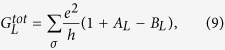



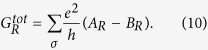



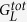
 and 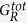 shown in Fig. 5 in the condition of *α*_2_ = 0 are obtained by Eqs (9) and (10). It is found that there also exists a bias voltage window, corresponding to the above-mentioned energy window. In the bias voltage window 0 < *eV* < 2*α*_1_, the conductances in the left and right leads at *h*_0_ = 0 are the same irrespective of the magnetic configurations. This is natural and indicates that the current is completely contributed from the nonlocal AR. Nevertheless, for *h*_0_ ≠ 0, the conductances not only in the left and right leads but also in the F and AF alignments are much different. In the corresponding bias voltage window, although the conductances in the F alignments exhibit the same behaviors as those for *h*_0_ = 0, at the resonant points, the normal reflection is totally suppressed and the conductance is just *e*^2^/*h* in each spin channel and 2*e*^2^/*h* in total in the left lead while the conductance is only *e*^2^/*h* in total in the right lead . These can be explained as follows: For the spin-up channel, the conductance is completely contributed from the nonlocal AR and there is always an electron going into the SC from the right when an electron is coming from the left^15^. The current from spin-down channel is however totally suppressed, i.e, the current from the spin-up and -down channels after tunneling from the left to the right lead, flows only in the spin-up channel, therefore, the structure acts as a spin-filter. With the enhancement of *h*_0_, the bias voltage window also turns narrower and the number of the peaks reduces as well, which perfectly embodies characteristics of the nonlocal AR process. For the AF alignment, the conductances in both the left and right leads are 0 in the bias voltage window, which is much different from those for *h*_0_ = 0 and also exactly reflects the properties of four tunneling processes. The behaviors of the conductances beyond the bias voltage window similarly manifest the features of the the tunneling processes. The conductances stemming from the nonlocal AR and elastic cotunneling may cancel each other, thus the properties of the noise besides the conductance to probe the nonlocal AR process is also needed. The noise power *S*(*ω*) of the current in the junction, i.e., the Fourier transform of the autocorrelator 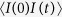 with *I*(0) and *I*(*t*) the currents at the time 0 and *t*, respectively, can be computed from the scattering formalism with the help of Wick’s theorem. The zero-frequency noise power can be obtained by^34^,^35^,^36^,^37^


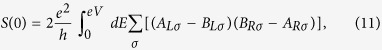


which can reflect the completing of all the processes and then the entanglement of quantum states. The noise response ∂*S*(0)/∂(*eV*) is shown in Fig. 6. For *h*_0_ = 0, the results in the F and AF alignments are naturally identical, which are the same as in ref. 15. In the bias voltage window 0 < *eV* < 2*α*_1_, the cross correlation is only contributed from the nonlocal AR and the normal reflection, therefore the noise response is always positive. At the resonant points, the fraction of the nonlocal AR is 100% and the noise response is 0 due to the absence of other competing processes. For the *h*_0_ unequal to 0, in the corresponding bias voltage window, the noise response is also always positive in the F alignments, while keeps zero in the AF alignments, indicating that all processes give no contributions to the noise response for both the incident spin-up and -down quasiparticles in the latter.

In summary, by the use of an extended BdG equation, we study the spin entangled state of the FI/*s*-wave SC/FI structure on a narrow QSH insulator strip, in which the interedge coupling is considered. The coupling brings about two conspicuous features. One is that, by tuning the gate voltage and exchange field of the two CF regions properly, in the energy window, the nonlocal AR probability in the F alignment for one spin always reach very high fraction, even up to 100%, while is thoroughly suppressed for the other opposite spin. And the other is that in the energy window, nonlocal AR probability in the AF alignment is zero and the currents for the left and right leads are both zero. The setup is therefore used as not only a spin filter with very high efficiency for entangled electron pairs but also a magnetic storage device with 100% efficiency. The tunnelling conductance and the noise power are presented as well. The device can be made in reality. The QSH system can be realized in mercury telluride quantum well. Due to the proximity effect of the FIs and SC here, the ferromagnetism and superconductivity are induced in the contacting areas of two edges. They may be usual FIs and SC, for instance, EuO for the former and Al for the latter. Only the transport properties of the two edges are involved, thus the quality of interfaces between the FI, SC, and QSH system cannot bring about significant effects.

## Additional Information

**How to cite this article**: Ji, T. T. *et al.* Coexistence of perfect spin filtering for entangled electron pairs and high magnetic storage efficiency in one setup. *Sci. Rep.* **6**, 24417; doi: 10.1038/srep24417 (2016).
